# Ultrasound-guided suprascapular nerve block with lidocaine vs. saline combined with physical exercises for the rehabilitation of supraspinatus tendinitis: a randomized double-blind controlled trial

**DOI:** 10.3389/fpain.2024.1490320

**Published:** 2024-11-12

**Authors:** Pericles Tey Otani, Roberto Del Valhe Abi Rached, Fabio Marcon Alfieri, Raymundo Soares de Azevedo Neto, Wu Tu Hsing, Linamara Rizzo Battistella, Marta Imamura

**Affiliations:** ^1^ Departamento de Ortopedia e Traumatologia, Hospital das Clinicas HCFMUSP, Faculdade de Medicina FMUSP, Universidade de Sao Paulo, Sao Paulo, Brazil; ^2^ Instituto de Medicina Fisica e Reabilitacao, IMREA, Hospital das Clínicas HCFMUSP, Faculdade de Medicina FMUSP, Universidade de Sao Paulo, Sao Paulo, Brazil; ^3^ Departamento de Patologia, Faculdade de Medicina da Universidade de Sao Paulo, FMUSP, Sao Paulo, Brazil; ^4^ Departamento de Medicina Legal, Bioetica, Medicina do Trabalho e Medicina Fisica e Reabilitacao, Faculdade de Medicina da Universidade de Sao Paulo, FMUSP, Sao Paulo, Brazil

**Keywords:** rotator cuff, rotator cuff injuries, supraspinatus tendinopathy, nervous block, pain management

## Abstract

**Introduction:**

Shoulder pain is the third leading cause of musculoskeletal complaints in primary care clinics. Its prevalence varies from 14% to 34%. Among all the structures that can cause shoulder pain, the most vulnerable to injury is the tendon of the supraspinatus muscle. The ideal management protocol is still unknown. To date, little is known in the literature about the use of ultrasound-guided suprascapular nerve block as a treatment for supraspinatus muscle tendinitis. Our objective was to assess the effects of the association of a single ultrasound-guided suprascapular nerve block combined with home-based rotator cuff exercises to reduce pain and improve shoulder functioning in patients with supraspinatus tendinitis.

**Methods:**

We evaluated the effect of a single ultrasound-guided suprascapular nerve block on pain and functioning of people with supraspinatus tendinitis. Diagnosis was performed using the positive Jobe test. Due to large disparity between clinical and radiological findings, only clinical diagnostic criteria were used to select patients. This was a double-blind, randomized, controlled, clinical study in which patients in the intervention group (*n* = 42) received a single injection of 5 ml of 2% lidocaine, while in the control group (*n* = 41) patients underwent the same procedure receiving saline solution 0.9%. All patients received face to face instructions by an experienced physiotherapist and a leaflet explaining home-based exercises. Pain and functioning were assessed using the Shoulder Pain and Disability Index (SPADI) questionnaire before the procedure, one week and 12 weeks after the procedure.

**Results:**

Patients in both groups improved significantly since the initial evaluation until the 12th week. Intervention group SPADI (pre, 1 week, 12 weeks): 75.80 ± 18.96, 56.25 ± 31.37, 46.31 ± 31.41 (*p* < 0.001); Control group SPADI: 75.49 ± 16.67, 50.51 ± 27.58, 49.37 ± 30.90 (*p* < 0.001). However, there were no significant differences between groups (*p* = 0.291).

**Discussion/conclusion:**

We concluded that both lidocaine and saline ultrasound-guided suprascapular nerve blocks reduce pain and improve shoulder functioning in patients with supraspinatus tendinitis. Unexpectedly, the same block performed with saline showed similar results and effects.

**Clinical Trial Registration:**

ClinicalTrials.gov, identifier [NCT02495818].

## 1 Introduction

Shoulder pain is the third leading cause of musculoskeletal complaints in primary care clinics, behind other prevalent complaints such as low back and knee pain (1, 2). The annual incidence rates varied from 7.7 to 62 per 1,000 person years (1). Its prevalence varies from 14% to 34% (3). Although 40% of the cases of shoulder pain are resolved in six months, after 12 months since the first pain episodes, around 50% of the patients remain with pain, despite undergoing traditional treatments (4). Shoulder pain may persist for one to three years after diagnosis in 20% and 14%, respectively (5). Rotator cuff tears account for 65%–70% of painful shoulders (3) and resulted in 272,148 surgeries in the United States in 2006 (6). Among all the structures that can cause shoulder pain, the most vulnerable to injury is the tendon of the supraspinatus muscle (7). Its impairment causes great pain when performing everyday activities, such as eating and dressing, as well as more complex work-related tasks (8).

The ideal management protocol is still unknown (9). Some interventions, such as physical therapy, exercises (10) and acupuncture (11) may require several weeks to improve symptoms (12). Surgical interventions are recommended in severe cases, however, they are expensive and require a period of post operatory immobilization (13). Moreover, indirect evidence from studies including several painful conditions highlight the disappointing results of oral medications (14) and physiotherapy (15) for managing chronic shoulder pain. Other interventions include extracorporeal shockwaves (16); platelet-rich plasma (17, 18); prolotherapy (18) and hypertonic dextrose injections (19–21). In the review by Lin et al. (19), the combination of extracorporeal shockwave therapy and physiotherapy was the only approach shown to improve both pain and shoulder function. SSNB improved mobility, only (16). The short duration of the analgesic effect of a local anesthetic nerve block can be enhanced by the use of continuous infusion of the anesthetics for weeks or the association of radiofrequency (15).

Suprascapular nerve block (SSNB) is often used to treat severe painful shoulder conditions, such as adhesive capsulitis (9), painful hemiplegic shoulder (9), after rotator cuff surgeries (22) and could be an alternative to treat other shoulder injuries (16, 23–25). The suprascapular nerve provides the sensory innervation of 70% of the shoulder (26). Therefore, the short duration of the effect of a local anesthetic block may allow for the performance of therapeutic exercises that acts in the physiopathology of the supraspinatus tendinitis (27). Suprascapular nerve block is superior to placebo injection of subcutaneous normal saline (23, 24) and convention physiotherapy (28, 29) to reduce pain and improve function in patients with non-specific shoulder pain. Ultrasound-guided suprascapular nerve block seems to be not superior to surface landmark-guided injections (25). However, the use of ultrasound for this procedure is an attractive choice due to its accessibility and portability and the fact that it does not expose patients, operators and doctors to radioactivity (30). Another advantage of using ultrasound is the fact that it allows real-time visualization of the procedure, which besides ensuring the safety of the procedure may also provide valuable diagnostic information about patients anatomy and the procedure as a whole (31). In fact, a meta-analysis (11 studies, 591 patients) evidenced the superiority of the suprascapular nerve block compared with placebo and physical therapy for treatment of chronic shoulder pain (15). However, no previous study assessed the effect of ultrasound-guided suprascapular nerve block for the management of supraspinatus muscle tendinitis, using a double-blind protocol.

Herein we used intervention with lidocaine as a treatment option for the supraspinatus muscle tendinitis (32). In the control group patients underwent the same procedure, but normal saline solution was used instead. Besides the intervention, patients were instructed by a physiotherapist to perform specific physical exercises at home and received printed material that explained how to perform them (27). Our objective was to assess the effects of the association of a single ultrasound-guided suprascapular nerve block combined with home-based rotator cuff exercises to reduce pain and improve shoulder functioning in patients with supraspinatus muscle tendinitis.

## 2 Materials and methods

This was a double-blind, randomized, controlled, clinical trial with parallel arms to investigate the efficacy of ultrasound-guided suprascapular nerve block combined with physical exercises in the treatment of tendinitis of the supraspinatus muscle, as assessed by pain and functionality parameters. The study was approved by the Research Ethics Committee of the Clinical Hospital of São Paulo through CAAE 12480513.7.0000.0068 and registered by clinicaltrials.gov under the identifier NCT02495818.

### 2.1 Patients and randomization

Randomization was generated by a computer program that created random blocks of four and six units. Occultation of randomization was maintained through opaque and sealed envelopes. On the day the patient arrived at our institute for the procedure, the research monitor at our center opened the envelope and instructed the nurse to prepare the syringe containing either lidocaine or saline, according to the randomization. The nurse prepared the appropriate syringe, labeled it with the patient's name, and sent it to the physician performing the procedure. The nurse did not disclose the content of the syringe, ensuring blinding of the physician to the administered substance. The patient, the physician, the physiotherapist and the statistician were blinded.

Patients with a clinical diagnosis of supraspinatus tendinitis were selected by the Jobe maneuver, justified by the fact that it is highly sensitive and specific (33). The radiological findings were not considered for including patients in the study due to the high clinical-radiological dissociation (34, 35).

#### 2.1.1 Inclusion criteria

Patients between 30 and 60 years of age, who had symptoms for a month or longer periods and had received a clinical diagnosis of tendinitis of the supraspinatus muscle established by a physiatrist or orthopedist by applying the Jobe maneuver (33, 36). Also, to be included in the study patients had to present a Visual Analog Scale (VAS) ≥4 in rest. We chose a smaller age range because supraspinatus tendinitis prevalence increases with age (37, 38), however, several other comorbidities could have influenced our results in case we included patients over 60 years due to the aging effects. All patients signed the informed consent form and were able to understand and answer the questions.

#### 2.1.2 Exclusion criteria

All patients who had any of the following symptoms, conditions or previous history were excluded: severe osteoarthritis of the shoulder, total rupture of the supraspinatus muscle, active systemic autoimmune disease, fracture of the humerus, fracture of the acromion or clavicle, shoulder dislocation or subluxation, any disease that could lead to spasticity of the upper limbs, such as quadriplegia or stroke, systemic changes that could induce peripheral neuropathy, such as decompensated diabetes or thyroid disease, severe cervical injury previously diagnosed by nuclear magnetic resonance, such as cervical disc herniation or stenosis spinal disorders, which resulted in motor disorders, allergy or hypersensitivity to topical or systemic anesthetics, use of oral or subcutaneous anticoagulation, blood dyscrasias, fibromyalgia according to the 1990 American College of Rheumatology criteria, psychiatric illnesses not controlled or treated by two or more medications, acute or chronic renal failure, hypoxemic respiratory pathologies such as Chronic Obstructive Pulmonary Disease or pulmonary fibrosis, arrhythmias (except isolated supraventricular extrasystoles), coronary insufficiency or heart failure class 2 or higher, pregnant women and patients who could not comply with treatment due to social factors. It is natural and intuitive that myofascial pain syndrome coexists with rotator cuff syndrome, since there is a local inflammatory process and muscle strength imbalance with areas of overload and weakness, which act as trigger points activators (39). Therefore, it was not considered as an exclusion criterion.

#### 2.1.3 Recruitment

Patients who had received medical care or were on the waiting list for rehabilitation treatment at the Lucy Montoro Rehabilitation Medicine Institute in São Paulo, Brazil, and who had a clinical diagnosis of rotator cuff syndrome, as identified in the medical records by the ICD-10 (International Classification of Diseases) code M75.1, were invited for an initial evaluation.

Social agents were responsible for contacting the patient. They applied a quick questionnaire about pathology, age, place of residence and explained the treatment. Patients interested in participating in the study were invited to attend our institution, where they received an explanation about the study and its procedures, including possible complications. Upon agreeing, they signed the informed consent form.

#### 2.1.4 Sample size calculation

To calculate the sample size, we considered the Shoulder Pain and Disability Index (SPADI) questionnaire from a previous published clinical trial performed in patients with a diagnosis of shoulder pain, submitted to a suprascapular nerve block not guided by ultrasound (23). We used this data because there was no similar study in the literature at that time.

The sample size calculation was based on the mean variation (delta) between two independent groups. Assuming a significance level (alpha) of 0.05 (two-tailed test), a statistical power of 80%, and data from the SPADI questionnaire used in this study, the following parameters were applied: the mean delta in the intervention group over 12 weeks was 13.5, and in the control group, 2.6. The standard deviation of the delta in the intervention group was 19.3, and in the control group, 17.4. Based on these values, the calculated sample size was 45 patients per arm. To account for an anticipated 20% loss to follow-up, 11–12 additional patients were included in each group, leading to a total of 113 participants.

### 2.2 Intervention

A Siemens Sonoline G40 ultrasound device and a linear transducer were used for the ultrasound-guided suprascapular nerve block, with adjustments according to each patient anatomy (40).

The patient was placed in a sitting position with the hand ipsilateral to the pain resting on the contralateral shoulder. The doctor was positioned behind the patient. Antisepsis of the region was performed with chlorhexidine solution. A thin layer of sterile gel was placed between the ultrasound transducer and the skin. An initial ultrasound evaluation was performed to guide the procedure. Then, the transducer was placed parallel to the spine of the scapula, it was moved superiorly towards the supraspinatus fossa, and then laterally until the scapular notch, where the suprascapular nerve crosses. The nerve is visualized as a round, hyperechoic structure, on average 4–5 cm in depth and lies just below the transverse ligament of the scapula.

**Figure 1:**
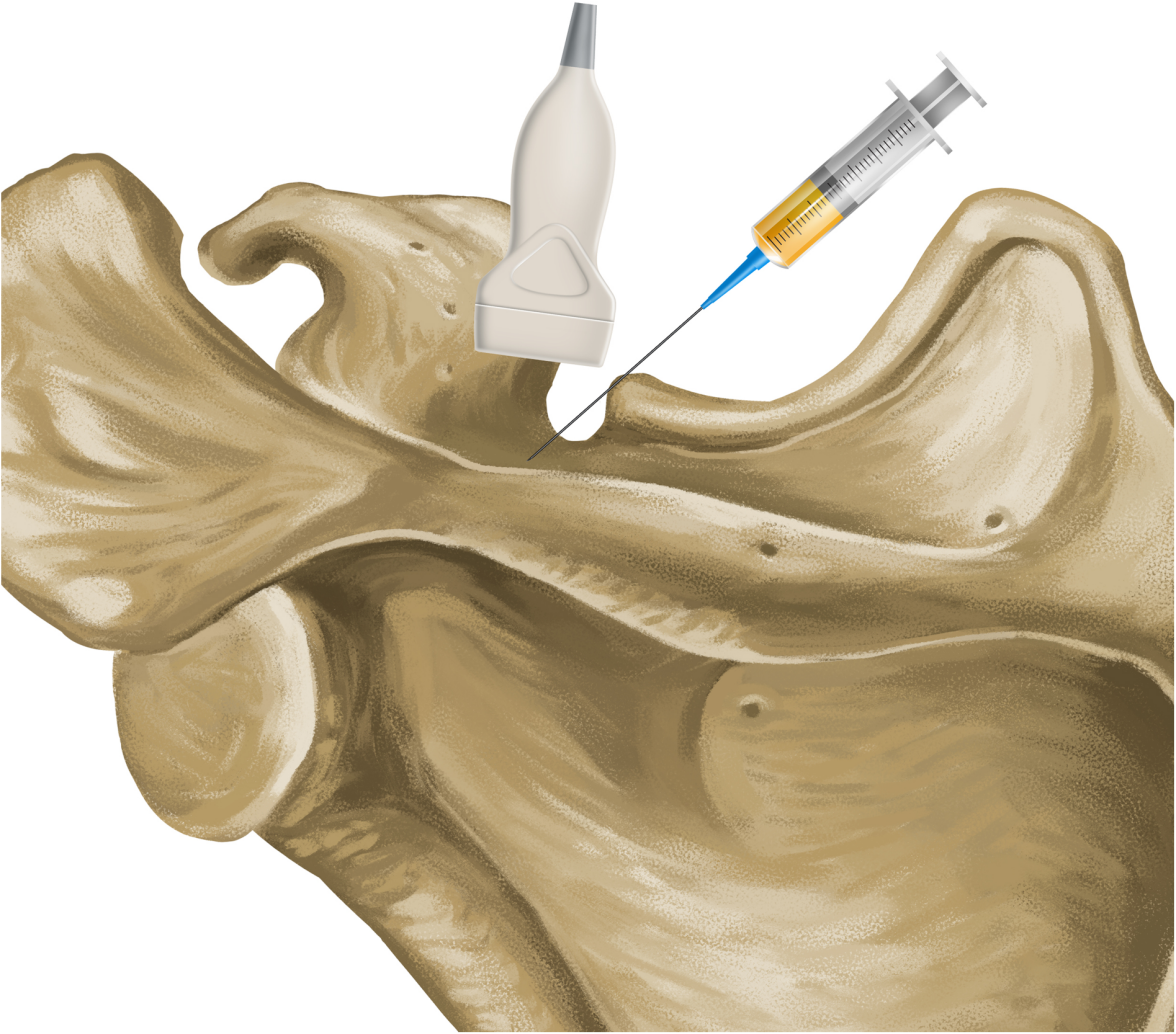
Anatomical relationship between the suprascapular notch, ultrasound transducer and needle.

**Figure 2:**
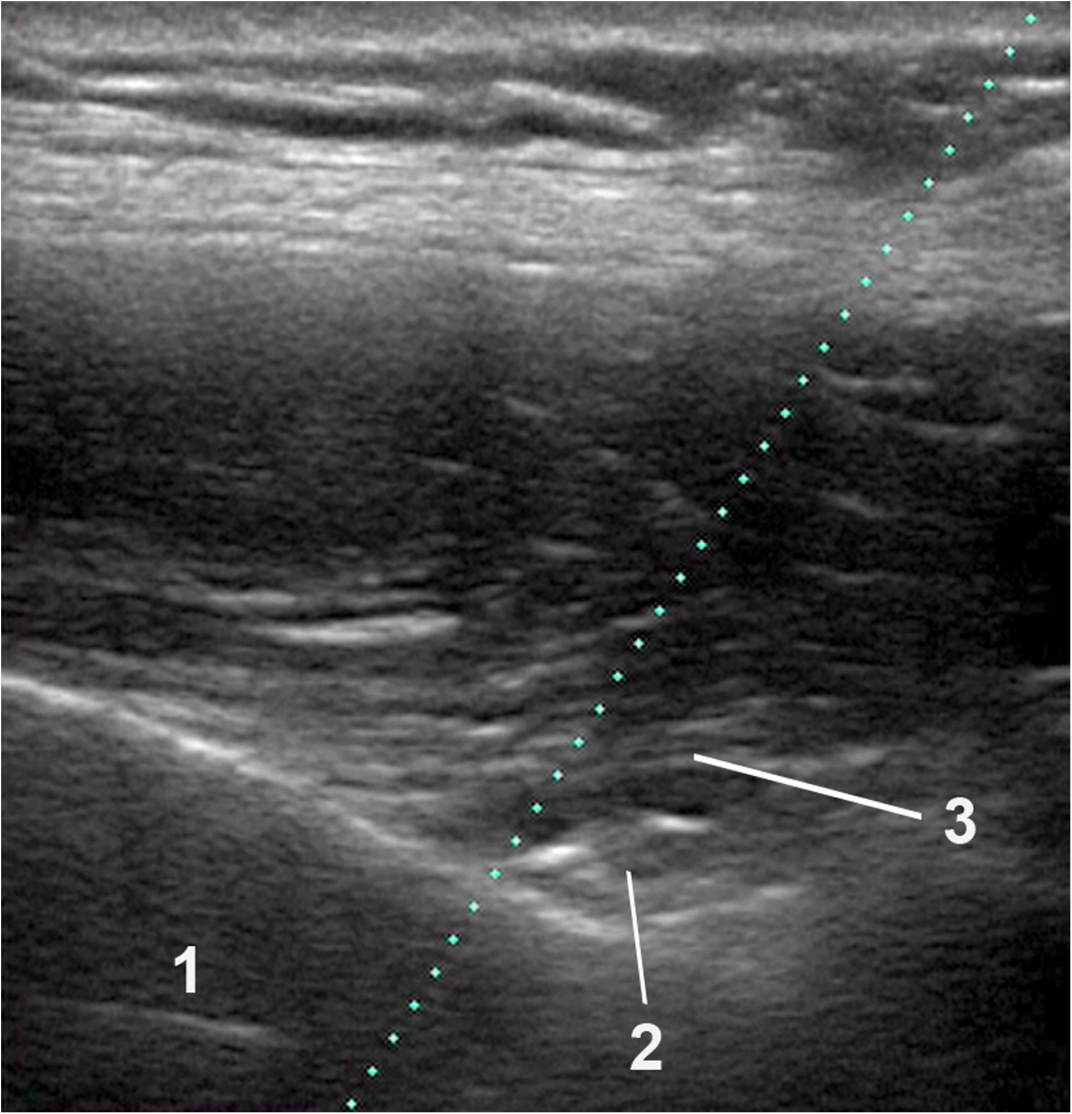
Ultrasound imaging anatomy. Caption: (1) Scapula. (2) Suprascapular nerve. (3) Transverse ligament of the scapula.

**Figure 3:**
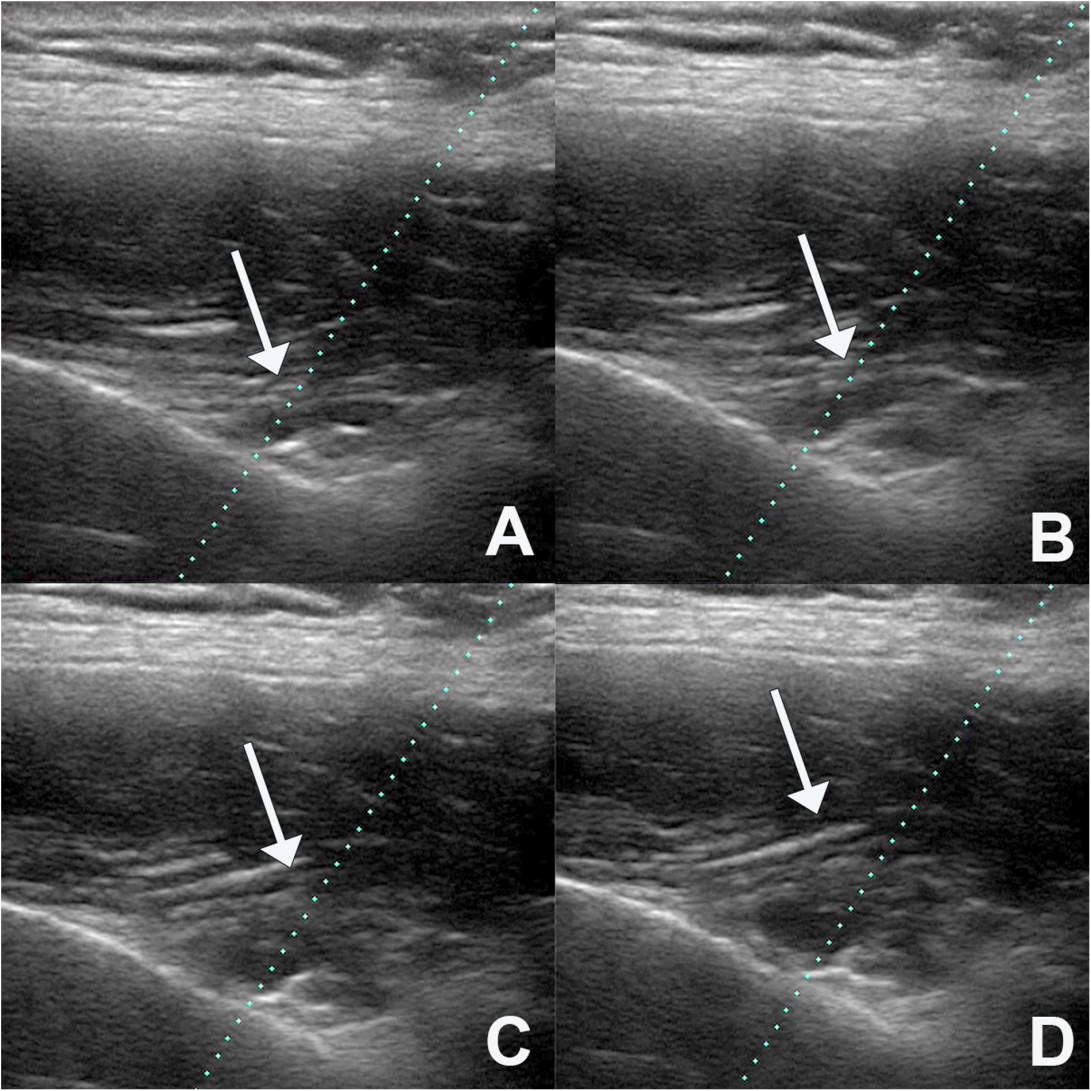
Expansion of the injected fluid, showing that it progressively elevates the transverse ligament of the scapula **(A–D)**.

The needle used was 10 cm long, 22G thick and had a Quincke tip. It was inserted along the longitudinal axis of the transducer (Figures 1, 2). It was slowly deepened by adjusting the entry angle and the needle path so that 5 ml of 2% lidocaine could be injected close to the suprascapular nerve, in the notch of the scapula. The needle was visualized in its entirety, as well as the injection and local expansion of the anesthetic (Figure 3). Typically, electrical stimulation is employed to locate nerves by eliciting corresponding muscle movements. However, in our study, the nerve was visualized directly using ultrasound imaging, rendering electrical stimulation unnecessary. The patient was observed for 1 h. If there were no complications, the patient was discharged and returned to the outpatient clinic the following week for a new evaluation.

Patients allocated to the control group received a procedure similar to the intervention group, but instead of lidocaine, they received saline. Neither the patients nor the physician performing the procedure were aware of the substance being administered, as they received a syringe from the nursing staff that was labeled only with the patient's name, without any indication of its contents.

In addition to the procedure, all patients received conservative treatment, which included instructions for home exercise and the use of medication as needed for pain management. The prescribed exercises were introduced by a physical therapist during the first follow-up appointment, one-week post-intervention. The Codman pendulum movements and Hughston exercises were selected due to their simplicity and ease of comprehension (27). Each patient received an instructional booklet containing illustrative figures and detailed educational text. Patients were advised to perform two sets of 12 repetitions for each exercise daily at home. The exercise folder provided to the patients is available as Supplementary Material. If the patient experienced pain during treatment, paracetamol 500 mg every 8 h was indicated. Medications that patients were using prior to the study were not withdrawn.

### 2.3 Outcome measures

Pain and functioning were assessed using the Shoulder Pain and Disability Index (SPADI) in its validated Portuguese version, since the study was carried out in Brazil (8). Another assessment tool utilized was the Visual Analog Scale (VAS), which consisted of a horizontal line with the left endpoint labeled as “zero,” representing “no pain,” and the right endpoint labeled as “ten,” indicating “maximum pain.” The patient marked the pain level on this line during the evaluation after receiving an explanation. Questionnaires were applied immediately before the procedure, at one-week and 12-week follow-ups.

Pain threshold was evaluated by algometry in metamer structures related to supraspinatus tendinitis. Measurements were performed on the deltoid muscle, on the lateral region of the arm (parallel algometry using the “rolling pinch” technique) and on the interspinous ligament between the C5 and C6 vertebrae, which correspond, respectively, to the myotome, dermatome and sclerotome. Algometry of metameric structures is exploratory data, but may suggest effects of the procedure on central sensitization of pain in supraspinatus tendinitis. Pain threshold was defined as the minimum pressure needed to induce pain at each point in kg per cm^2^. Active and passive range of motion for shoulder flexion and abduction was assessed using goniometry. Both algometry and goniometry measures were evaluated immediately before the procedure, at one-week and 12-week follow-ups.

The primary endpoint was measured SPADI, while the secondary endpoint was measured by VAS, algometry and goniometry.

### 2.4 Statistical analysis

The software used for statistical analysis was JASP Team (2020) version 0.14.1 (University of Amsterdam). The mean SPADI scores of the intervention and control groups over time were compared using repeated measures analysis of variance (ANOVA). The missing data imputation was performed using the Last Observation Carried Forward (LOCF) method, where missing data were replaced by the last available observation, ensuring that all patients were included in the subsequent analyses, in line with the intention-to-treat principle. An interim analysis was pre-specified using the O'Brien-Fleming stopping boundary, with a stringent significance threshold set at a *p*-value of 0.01. This analysis was scheduled to take place at the midpoint of the study to assess whether early termination was warranted based on the accumulated data.

## 3 Results

### 3.1 Patients and randomization

A total of 186 patients were recruited in 3 years; 87 met the inclusion criteria for the screening visit, 4 dropped out for personal reasons before undergoing the procedure and were excluded from the study. The final study included 83 patients. Of these, 76 completed the third and final assessment. Five patients were evaluated twice, and two patients only completed the first assessment. The first patient was included in the study on July 19, 2013 and the last on June 16, 2015.

**Figure 4:**
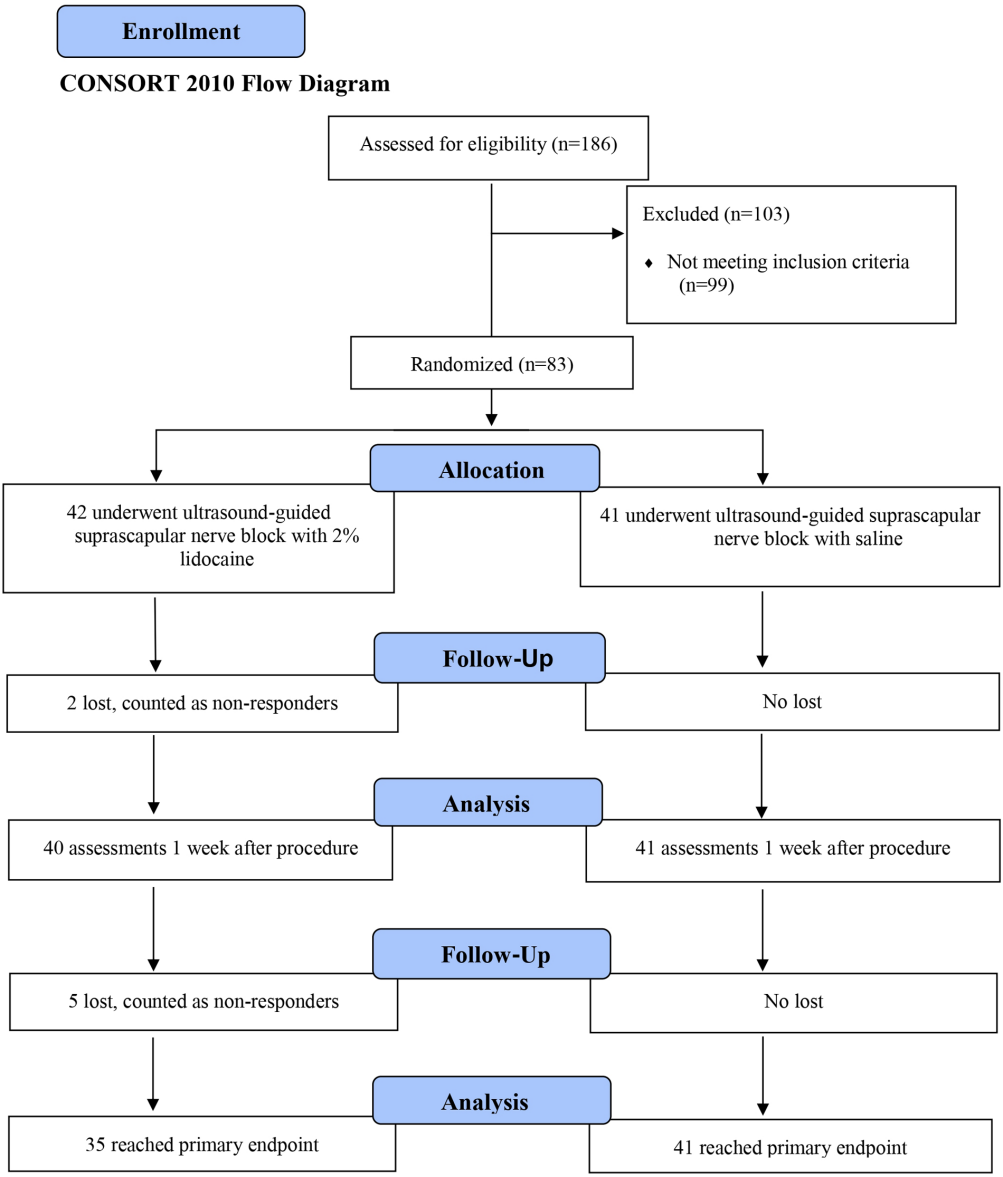
Flow diagram.

Table 1 presents the population demographics followed by the study flowchart in Figure 4.

**Table 1:** Population demographics and baseline assessments.

Demographics	Intervention	Control	*p*
Sample size: number of patients	42	41	
Age: average in years (SD)	47.43 (8.99)	49.78 (7.62)	0.20*
Pain duration in months: mean (SD)	46.95 (47.52)	56.44 (61.19)	0.43*
Right/left laterality (%)	27/15 (64/36)	28/13 (68/32)	0.70**
In physical therapy or physical activity at home: yes/no (%)	7/35 (17/83)	16/25 (39/61)	0.02**
In current use of pain medication: yes/no (%)	6/36 (14/86)	13/28 (32/68)	0.06**
Positive Pattey maneuver: yes/no (%)	31/11 (74/26)	37/4 (90/10)	0.05**
Positive Gerber maneuver: yes/no (%)	28/14 (67/33)	36/4 (90/10)	0.01**
Sex: male/female (%)	8/34 (19/81)	7/34 (17/83)	0.81**
Ethnicity: white/yellow/brown/black (%)	28/0/6/7 (68/0/15/17)	26/1/10/3 (65/2/25/8)	0.30**
Body mass index: mean (SD)	27.72 (4.81)	28.04 (4.54)	0.77*
Systemic arterial hypertension: yes/no (%)	7/35 (17/83)	13/28 (32/68)	0.11**
Diabetes Mellitus: yes/no (%)	7/35 (17/83)	3/38 (7/93)	0.19**
Smoking: yes/no (%)	4/38 (10/90)	11/30 (27/73)	0.04**
Visual analogue scale: mean (SD)	8.2 (1.28)	7.73 (1.47)	0.13*
Total SPADI: mean (SD)	75.57 (18.82)	75.49 (16.67)	0.98*
Algometry of the deltoid muscle in kg per cm^2^: mean (SD)	2.71 (1.05)	2.75 (0.83)	0.88*
Algometry of C6 subcutaneous in kg per cm^2^: mean (SD)	2.53 (1.00)	2.42 (0.85)	0.62*
Algometry of the C5–C6 interspinal ligament in kg per cm^2^: mean (SD)	2.48 (0.92)	2.45 (1.11)	0.91*
Passive shoulder abduction ROM in degrees: mean (SD)	99.39 (25.50)	112.80 (32.33)	0.04*
Passive shoulder flexion ROM in degrees: mean (SD)	110.49 (30.51)	114.27 (30.03)	0.57*
Active shoulder abduction ROM in degrees: mean (SD)	76.83 (21.50)	85.24 (24.19)	0.10*
Active shoulder flexion ROM in degrees: mean (SD)	78.29 (21.70)	81.93 (22.21)	0.46*

SD, standard deviation; %, percentage; VAS, visual analogue scale; *SPADI*, shoulder pain and disability index; ROM, range of motion; kg, kilogram; cm, centimeter.

* *t* test.

** Qi square test.

#### 3.1.1 Primary endpoint

**Figure 5:**
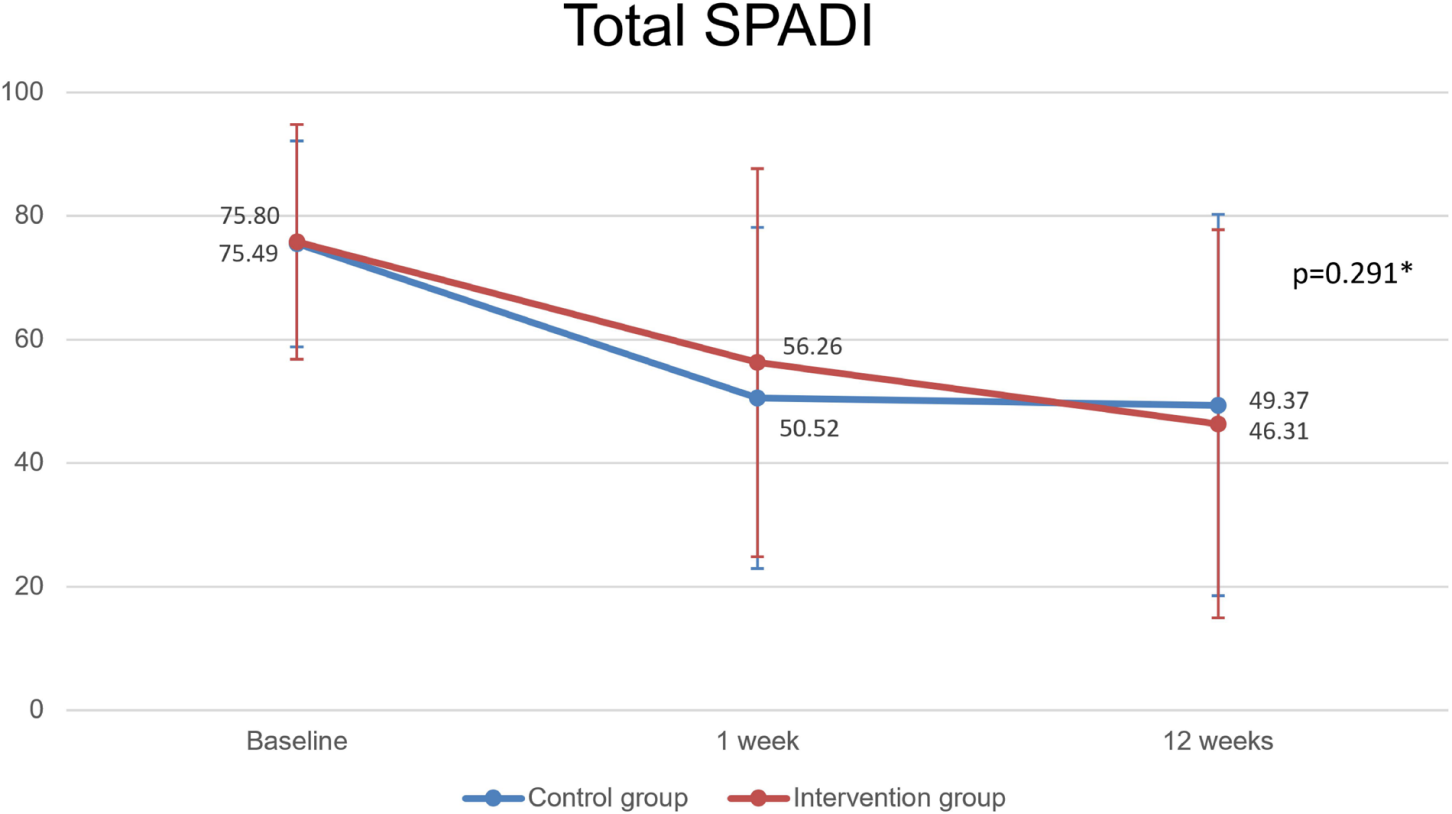
Primary outcome: total SPADI parameter. Caption: SPADI, shoulder pain and disability index; (*): Repeated measures ANOVA.

The ANOVA test with repeated measures was used to assess the interaction of the intervention over time for the primary outcome, SPADI, using the JASP Team 2020 software version 0.14.1. This statistical analysis is described in Figure 5; Tables 2, 3.

**Table 2:** Type III Sum of squares for SPADI adjusted for physical therapy, pain medications and passive shoulder abduction range of motion.

Source	Sphericity correction	Sum of squares	df	Mean square	F-value	*p*-value
SPADI	Greenhouse-Geisser	699.58	1.94	360.28	1.06	0.35
SPADI ✻ Intervention type	Greenhouse-Geisser	2,765.22	1.94	1,424.05	4.21	0.02
SPADI ✻ In physical therapy or physical activity at home	Greenhouse-Geisser	239.99	1.94	123.59	0.37	0.69
SPADI ✻ In current use of pain medication	Greenhouse-Geisser	150.21	1.94	77.36	0.23	0.79
SPADI ✻ Passive shoulder abduction ROM	Greenhouse-Geisser	1,628.69	1.94	838.75	2.48	0.09
SPADI ✻ Intervention type ✻ In physical therapy or physical activity at home	Greenhouse-Geisser	813.34	1.94	418.86	1.24	0.29
SPADI ✻ Intervention type ✻ In current use of pain medication	Greenhouse-Geisser	553.52	1.94	285.06	0.84	0.43
SPADI ✻ In physical therapy or physical activity at home ✻ In current use of pain medication	Greenhouse-Geisser	72.92	1.94	37.55	0.11	0.89
SPADI ✻ Intervention type ✻ In physical therapy or physical activity at home ✻ In current use of pain medication	Greenhouse-Geisser	507.46	1.94	261.33	0.77	0.46
Residuals	Greenhouse-Geisser	47,961.8	141.75	338.35		

Legend: *SPADI*, shoulder pain and disability index; ROM, range of motion.

**Table 3:** Primary and secondary endpoint.

Primary endpoint	Mean ± SD (Intervention)	Mean ± SD (Control)	*p* *
SPADI			
Baseline	75.80 ± 18.96	75.49 ± 16.67	
1-week post-intervention	56.25 ± 31.37	50.51 ± 27.58	0.291
12-weeks post-intervention	46.31 ± 31.41	49.37 ± 30.90	
*p*-value	<0.001	<0.001	
Secondary endpoint	Mean ± SD (Intervention)	Mean ± SD (Control)	*p* *
VAS			
Baseline	8.20 ± 1.28	7.73 ± 1.47	
1-week post-intervention	5.57 ± 3.13	4.50 ± 2.55	0.126
12-weeks post-intervention	4.61 ± 3.33	4.95 ± 3.43	
*p*-value	<0.001	<0.001	
Active shoulder abduction ROM (degrees)			
Baseline	76.83 ± 21.50	85.24 ± 24.19	
1-week post-intervention	93.76 ± 36.27	97.90 ± 30.99	0.140
12-weeks post-intervention	103.66 ± 42.65	95.78 ± 42.20	
*p*-value	<0.001	<0.001	
Passive shoulder abduction ROM (degrees)			
Baseline	99.39 ± 25.50	112.80 ± 32.33	
1-week post-intervention	122.80 ± 39.82	127.73 ± 33.49	0.010
12-weeks post-intervention	128.10 ± 42.23	119.46 ± 38.85	
*p*-value	<0.001	<0.001	
Active shoulder flexion ROM (degrees)			
Baseline	78.29 ± 21.70	81.93 ± 22.21	
1-week post-intervention	92.98 ± 38.32	109.76 ± 40.46	0.006
12-weeks post-intervention	109.88 ± 40.63	97.44 ± 46.19	
*p*-value	<0.001	<0.001	
Passive shoulder flexion ROM (degrees)			
Baseline	110.49 ± 30.51	114.27 ± 30.03	
1-week post-intervention	126.73 ± 35.69	137.56 ± 37.80	0.028
12-weeks post-intervention	135.71 ± 34.29	127.10 ± 36.57	
*p*-value	<0.001	<0.001	
Algometry of the deltoid muscle (kg per cm^2^)			
Baseline	2.71 ± 1.05	2.75 ± 0.83	
1-week post-intervention	4.73 ± 2.81	4.29 ± 2.08	0.732
12-weeks post-intervention	4.53 ± 3.07	4.44 ± 3.20	
*p*-value	<0.001	<0.001	
Algometry of the C5–C6 ligament (kg per cm^2^)			
Baseline	2.48 ± 0.92	2.45 ± 1.11	
1-week post-intervention	3.85 ± 2.37	3.36 ± 1.50	0.564
12-weeks post-intervention	3.56 ± 2.31	3.25 ± 2.04	
*p*-value	<0.001	<0.001	
Algometry of the C6 subcutaneous (kg per cm^2^)			
Baseline	2.53 ± 1.00	2.42 ± 0.85	
1-week post-intervention	3.46 ± 1.98	3.09 ± 1.29	0.718
12-weeks post-intervention	3.49 ± 2.54	3.09 ± 1.88	
*p*-value	<0.001	<0.001	

Legend: SD, standard deviation; VAS, visual analogue scale; SPADI, shoulder pain and disability index; ROM, range of motion; kg, kilogram; cm, centimeter.

* Repeated measures ANOVA.

Patients in both groups improved significantly since the initial evaluation until the 12th week. Intervention group SPADI (pre, 1 week, 12 weeks): 75.80 ± 18.96, 56.25 ± 31.37, 46.31 ± 31.41 (*p* < 0.001); Control group SPADI: 75.49 ± 16.67, 50.51 ± 27.58, 49.37 ± 30.90 (*p* < 0.001); however, with no significant differences between groups (*p* = 0.291).

Medication use and physical activity at home was unbalanced within the two groups at baseline. Adjusted statistical analysis revealed that these factors have not influenced or results. The ANOVA for repeated measures for SPADI, considering the combined effect of intervention group, physical therapy/exercise and pain medication, showed no significant difference among the resulting subgroups over time (*p* > 0.05). Results are described in Table 2.

#### 3.1.2 Secondary endpoint

For exploratory and investigative purposes only, the groups of VAS, goniometry and algometry variables were compared using the ANOVA test with repeated measures using the JASP Team 2020 software version 0.14.1.

**Figure 6:**
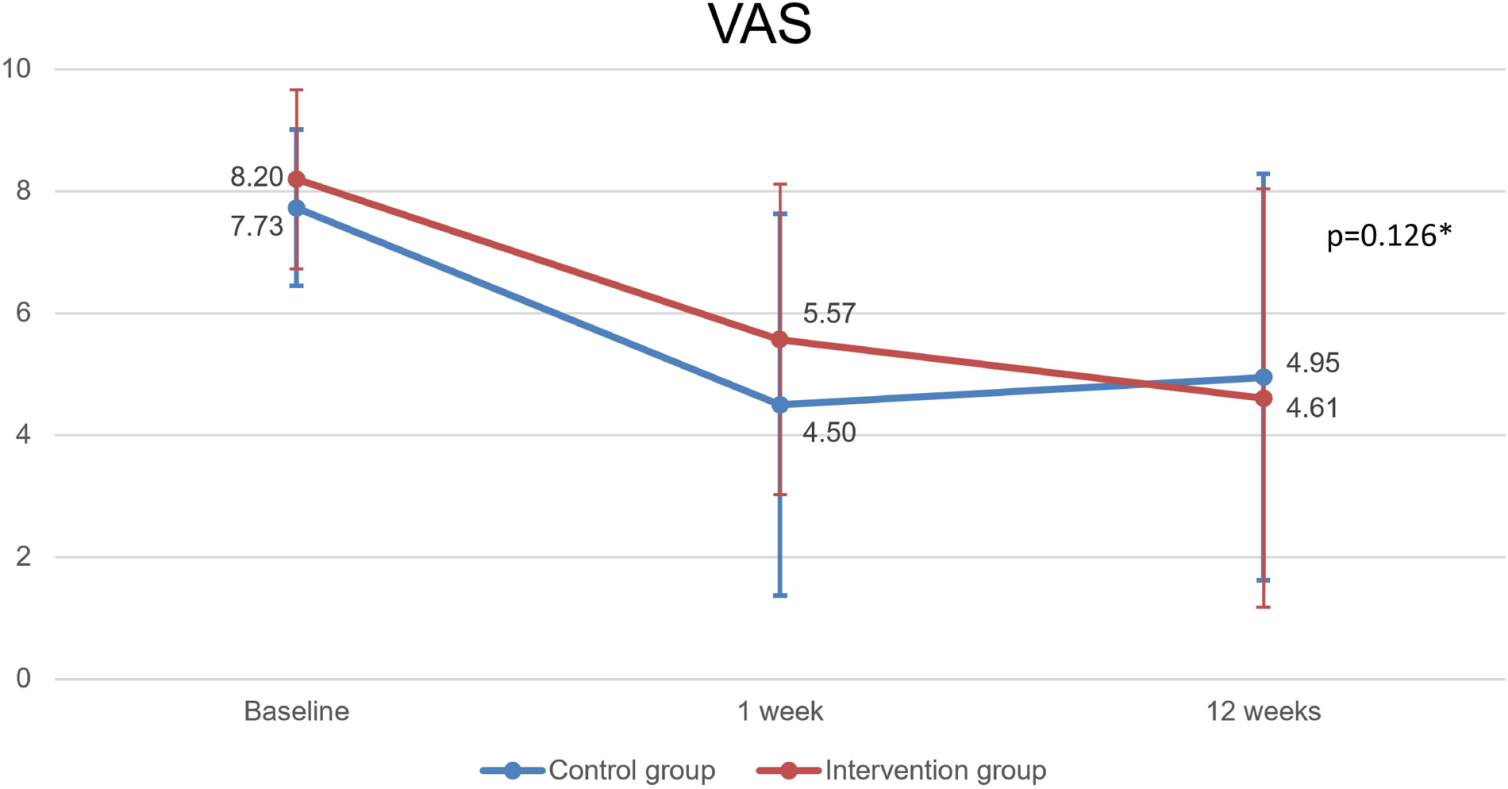
VAS parameter. Caption: VAS, visual analogue scale; (*): Repeated measures ANOVA.

Patients in both groups improved significantly since the initial evaluation until the 12th week. Intervention group VAS (pre, 1 week, 12 weeks): 8.20 ± 1.28, 5.57 ± 3.13, 4.61 ± 3.33 (*p* < 0.001); Control group: 7.73 ± 1.47, 4.50 ± 2.55, 4.95 ± 3.43 (*p* < 0.001). However, with no significant differences between groups (*p* = 0.126). The results are summarized in Figure 6; Tables 3, 4.

**Table 4:** Type III Sum of squares for VAS adjusted for physical therapy, pain medications and passive shoulder abduction range of motion.

Source	Sphericity correction	Sum of squares	df	Mean square	F-value	*p*-value
VAS	Greenhouse-Geisser	17.96	1.89	9.48	1.96	0.15
VAS ✻ Intervention type	Greenhouse-Geisser	58.8	1.89	31.05	6.42	2.57 × 10^−3^
VAS ✻ In physical therapy or physical activity at home	Greenhouse-Geisser	2.43	1.89	1.28	0.27	0.76
VAS ✻ In current use of pain medication	Greenhouse-Geisser	34.86	1.89	18.41	3.81	0.03
VAS ✻ Passive shoulder abduction ROM	Greenhouse-Geisser	9.61	1.89	5.08	1.05	0.35
VAS ✻ Intervention type ✻ In physical therapy or physical activity at home	Greenhouse-Geisser	19.44	1.89	10.26	2.12	0.13
VAS ✻ Intervention type ✻ In current use of pain medication	Greenhouse-Geisser	25.56	1.89	13.5	2.79	0.07
VAS ✻ In physical therapy or physical activity at home ✻ In current use of pain medication	Greenhouse-Geisser	20.88	1.89	11.02	2.28	0.11
VAS ✻ Intervention type ✻ In physical therapy or physical activity at home ✻ In current use of pain medication	Greenhouse-Geisser	4.67	1.89	2.47	0.51	0.59
Residuals	Greenhouse-Geisser	668.2	138.25	4.83		

Legend: VAS, visual analogue scale; ROM, range of motion.

Medication use and physical activity at home was unbalanced within the two groups at baseline. Adjusted statistical analysis revealed that these factors have not influenced or results. The ANOVA for repeated measures for VAS, considering the combined effect of intervention group, physical therapy/exercise and pain medication, showed no significant difference among the resulting subgroups over time (*p* > 0.05). Adjustments are described in Table 4.

Some subjects exhibited noteworthy results. At the first-week post-procedure assessment, three patients in the intervention group and none in the control group reported a SPADI score of zero, while three patients in the intervention group and three in the control group recorded a VAS score of zero. At the 12-week post-procedure assessment, five patients in the intervention group and three in the control group had a SPADI score of zero, while four patients in the intervention group and six in the control group reported a VAS score of zero.

The Minimal Clinically Important Difference (MCID) for the Shoulder Pain and Disability Index (SPADI) is reported as 13.2 points (41). In our study, we observed mean reductions of 29.486 (SD: 26.511) in the lidocaine group and 26.121 (SD: 25.617) in the saline group, both significantly exceeding the threshold for clinical relevance. Similarly, for the Visual Analog Scale (VAS), the reported MCID is 1.37 points (42). The mean VAS reductions were 3.586 (SD: 3.116) in the lidocaine group and 2.780 (SD: 3.237) in the saline group, both surpassing the minimum clinically important change. Despite the lack of statistically significant differences between the groups, both procedures produced meaningful clinical improvements, with reductions approximately two to three times higher than the MCID for SPADI and VAS, respectively.

The magnitude of changes for each intervention was assessed by comparing pre-intervention and 12-week post-intervention scores, utilizing intragroup comparisons evaluated with a two-tailed paired *t*-test. G*Power software and Cohen's *d* statistic were used to calculate effect sizes. For the intervention group, the SPADI and VAS scores demonstrated large effect sizes, with *d* = 1.07 and *d* = 1.06, respectively. Similarly, in the control group, SPADI and VAS scores yielded effect sizes of *d* = 0.97 and *d* = 0.93, respectively. Given that Cohen's *d* values above 0.8 indicate a large effect, both the lidocaine and saline interventions may offer clinically beneficial effects in the treatment of patients.

#### 3.1.3 Adverse events

Adverse events are described in Table 5.

**Table 5:** Adverse events.

Adverse events (sample size)	Intervention (42)	Control (41)	*p*
Local pain: yes/no (%)	6/36 (14.3/85.7)	2/39 (4.9/95.1)	0.265*
Temporary loss of strength: yes/no (%)	1/41 (2.4/97.6)	0/41 (0/100)	0.999*
Temporary loss of sensitivity: yes/no (%)	4/38 (9.5/90.5)	1/40 (2.4/97.6)	0.360*
Nausea and vomiting: yes/no (%)	1/41 (2.4/97.6)	2/39 (4.9/95.1)	0.616*
Local hematoma: number of patients	None	None	

* Fisher's exact test.

### 3.2 Interim analysis and statistical power

The interim analysis was performed after data collection from 83 patients. Although the significance threshold of *p*-value < 0.01 was not met, the decision to terminate the study was based on several key considerations. First, the analysis showed that the collected data were sufficiently robust and consistent to allow for conclusive results. Specifically, the statistical power of the study reached 92.2% for the total SPADI and 95.6% for the VAS, suggesting a high probability that the outcomes would remain consistent with those of a full sample size. Given this high level of statistical power and the strength of the evidence, continuing the study was deemed ethically unjustifiable, as it would involve exposing additional patients to an experimental treatment. Consequently, based on the interim analysis, the study was discontinued early, adhering to the pre-specified stopping criteria and ensuring both scientific rigor and ethical responsibility.

## 4 Discussion

In this study, a single ultrasound-guided suprascapular nerve block combined with home-based rotator cuff exercises improved pain and functionality in people with supraspinatus tendinitis and this effect lasted for at least 12 weeks. This method is usually used in some severe shoulder pathologies, such as adhesive capsulitis (9), painful shoulder of the hemiplegic (9) and after shoulder surgery (22). In addition to these conditions, supraspinatus tendinitis can also compromise shoulder functionality, leading to central sensitization and chronic pain (43). So that, suprascapular nerve block could be considered as an alternative treatment. This method has few adverse events and can be easily included in routine consultations. Our hypothesis is that conventional interventions, such as medication and physiotherapy, could be associated with this procedure to enhance results, which are so desired in difficult-to-treat pathologies.

We have demonstrated that this nerve block can be faster and have immediate results when compared to physical therapy (44) and acupuncture (45). A rotator cuff syndrome rehabilitation exercise program typically lasts from 3 weeks to 6 months, depending on the severity of the injury (44). It is described in the literature that from the fourth week onwards, physical therapy may have a similar effect to some procedures, such as corticosteroid injections, in the treatment of rotator cuff syndrome (46). Sonune (47) demonstrated that the improvement in pain and range of motion with suprascapular nerve block was superior when compared to intra-articular corticosteroid injection on the first day after the procedure, suggesting that, anesthetics should be considered as the preferred substance whenever possible in relation to corticosteroids, as they present faster results and have fewer contraindications and side effects. In Favejee's systematic review (48), the effects of suprascapular nerve block were found to be superior to those of acupuncture in terms of pain reduction and improvements in range of motion, evaluated 30 min after the procedure. Although this data pertains specifically to adhesive capsulitis, it is reasonable to consider that some degree of extrapolation to supraspinatus muscle tendinitis may be applicable.

Suprascapular nerve block becomes important as a possibility to be considered prior to rotator cuff surgical repair in the initial approach. US-guided suprascapular nerve block is not as expensive as surgery (49), which makes it feasible for daily consultations. The biggest cost is the doctor's time, as the health service usually has the ultrasound device. Routine supplies such as syringe, needle, anesthetic, ultrasound gel and gloves are inexpensive. The advantages over surgery are numerous, from low cost (49) to lower risk of complications as it is a less invasive procedure (50). The most common surgery adverse event is failure of tendon healing following rotator cuff repair, which may result in a further procedure (51). Post-surgical infection (51), palsies due to nerve damage (51), as well as post anesthesia cerebrovascular event may be also reported (52).

Our results confirm previous findings from Ryosa's meta-analysis (50) who illustrated that conservative treatment can sometimes have a similar outcome compared to surgical treatment of the rotator cuff tear. Moosmayer (53) and Lambers (54) found better results in favor of surgical treatment, however, the differences were small and, according to the authors themselves, may not represent clinical importance. Despite the shorter follow-up period in our evaluation, we concur with the findings of Kukkonen (55), who reported that physical therapy and surgery may not differ in terms of pain and functionality, as measured by the Constant Score, over the course of one year in patients with supraspinatus tendon injuries.

It is well known that exercise and pain medications can improve pain and function specially in the acute phase of patients suffering from shoulder pain. Our original insight was that the pain relief achieved by the ultrasound-guided suprascapular nerve block allowed patients to perform their home exercises which allowed for the three-month results observed in our study. We have not anticipated that the saline injection would provide similar improvements in the mid-term follow-up.

On the other hand, it cannot be excluded that normal saline may have independently acted as a perineural space expander, without the involvement of local anesthetic, anti-inflammatory, or nerve repair mechanisms (56). Considering the mechanism of hydrodissection, normal saline may also be considered an injectable for this purpose (56). We cannot rule out the potential mechanical effect of hydrodissection that normal saline may have provided to patients randomized to the control group, potentially facilitating nerve release and improving its gliding under the transverse scapular ligament.

We should also consider that we have only included chronic patients with a mean duration of 46.95 (SD: 47.52) months. It is known that medication and exercises have limited effect for these chronic patients (57). Chronic and long-term use of analgesics, opioids and anti-inflammatory medications should be considered with caution due to their potential side effects and harm. Low adherence to home exercises is also a challenge in the management of chronic musculoskeletal pain conditions (58). In any case, future studies should carefully monitor medication intake and adherence to the home exercise regime. We have included both interventions as the local ethical committee would not allow a group with placebo intervention only.

Part of the placebo effect can be attributed to the Hawthorne effect, in which there is a change in patient's behavior due to the care and attention received during treatment (59, 60). Patients were evaluated by a variety of professionals during the study, including physicians, physical therapists, nurses and social workers, and received care before, during and after the procedure at a renowned institution for a period of three months. All the affection dedicated to the patient may have influenced the response (59, 60). It is described in the literature that placebos present better results as procedures than as oral medications, as they are more invasive (61).

In addition to the psychological and behavioral aspects, the mechanical effect of the procedure, which consists of placing the needle in the patient and injecting the saline solution, can also treat pain (23). Acupuncture and dry needling present consistent results in the literature and may explain part of the response of any procedure that involves needle placement (11, 45, 62). There is a certain therapeutic effect due to local micro bleeding and disruption of the cell membrane, which triggers the beginning of an inflammatory cascade (63). This acute inflammation can lead to tissue repair and growth, with consequent clinical recovery (63). With deep needling and expansion of local fluid, mechanoreceptors of more structures are stimulated, which may contribute to a greater effect (64).

In the 1980s, Mayer (65) demonstrated that the use of naloxone, an opioid antagonist, in high doses could block the needling effect, suggesting analgesia induced by mechanical rather than chemical stimuli. Another study suggested that the endocannabinoid system could also be activated by the dry needling stimuli and may have contribute to pain control (66). Thus, several mechanisms can explain how this procedure works. However, more studies are needed to better understand the reason for the improvement of pain and functioning even in the saline group.

An important factor identified in our study, improvement is pain and shoulder function were statistically significant from baseline data and approximately two times superior than the minimal clinically important difference (MCID), both for SPADI and VAS. Our findings highlight the clinical relevance of the combination of SSNB with home exercises. Previous findings, compiled in a recent systematic review, did not demonstrate significant improvements in SPADI and VAS scores for patients with chronic shoulder pain following SSNB (16). Only mobility improved. Results similar to ours were only identified with the combination of extracorporeal shockwaves and conventional physiotherapy (16). A recent randomized controlled trial compared the effectiveness of ultrasound guided vs. landmark-guided suprascapular nerve block for chronic shoulder pain also using SPADI and VAS as outcome measures. Improvement in pain and shoulder function was similar than those obtained in our study.

Similar to the increase in mobility demonstrated previously (16), we have also identified a significant and superior improvement in passive should abduction, passive and active shoulder flexion in our patients. As a secondary outcome, this is an exploratory finding that should be better explored in future studies. As the body of scientific evidence increases, better understanding of the mechanisms involved on the pathophysiology of complex shoulder conditions will be also elucidated. Improvement in the ROM maybe indirect evidence that patients performed their home exercise regime. Future studies should further investigate the effects of SSNB on the pathophysiology of chronic supraspinatus tendinitis.

Interesting to highlight, is the low attrition observed in our study. The majority of the patients (91.6%) returned for the 12-week evaluation. Another striking benefit was the lack of severe and long-lasting adverse events or complications with the method. Although the absolute numbers of adverse events tended to be higher, local pain, temporary loss of strength and sensitivity and nausea and vomiting were not statistically significantly more frequent in the lidocaine injection group compared with the saline.

### 4.1 Study limitations

Medication use and physical activity at home was unbalanced within the two groups at baseline. Adjusted statistical analysis revealed that these factors have not influenced our results. On the other hand, a major limitation of our study was the lack of appropriate monitoring of adherence and/or rate at which medication intake and home exercise were carried out during the follow-up period. We have only given face to face instructions of the home exercise regime and given a booklet containing all the exercises. However, we have only included chronic patients with a mean duration of 46.95 (SD: 47.52) months. Medication and exercise have limited effect in these chronic patients (14, 15). Despite no control, we have observed a significant improvement since baseline for both groups. It is important to acknowledge that sex and age may have influenced our results and treatment response. However, due to statistical limitations regarding the number of variables that could be included in the repeated measures ANOVA for SPADI and VAS, we were unable to incorporate these variables in the adjusted analysis. No diagnosis of supraspinatus tendinitis was confirmed through imaging. The well-established clinical-radiological dissociation in the literature, though, supports the validity of clinical examination for diagnostic purposes (67). Other shoulder tendinitis as well as biceps injuries were not excluded, so there are no data on isolated rehabilitation of supraspinatus tendinitis. Another limiting factor is that the follow-up time was only three months, so there is no data on the actual duration of the intervention. On the other hand, this situation reflects the day-to-day of outpatient care.

## 5 Conclusion

Ultrasound-guided suprascapular nerve block with lidocaine combined with home-based rotator cuff exercises improved the patients’ pain and functionality over the 12 weeks of observation. Unexpectedly, the same block performed with saline showed similar results and effects.

## Data Availability

The original contributions presented in the study are included in the article/Supplementary Material, further inquiries can be directed to the corresponding author.
