# Supplementary figures and images for: Ultrasound-guided suprascapular nerve block with lidocaine vs. saline combined with physical exercises for the rehabilitation of supraspinatus tendinitis: a randomized double-blind controlled trial
Source: Front Pain Res (Lausanne). 2024 Nov 12;5:1490320. doi: 10.3389/fpain.2024.1490320 (PMC11588745; doi:10.3389/fpain.2024.1490320)

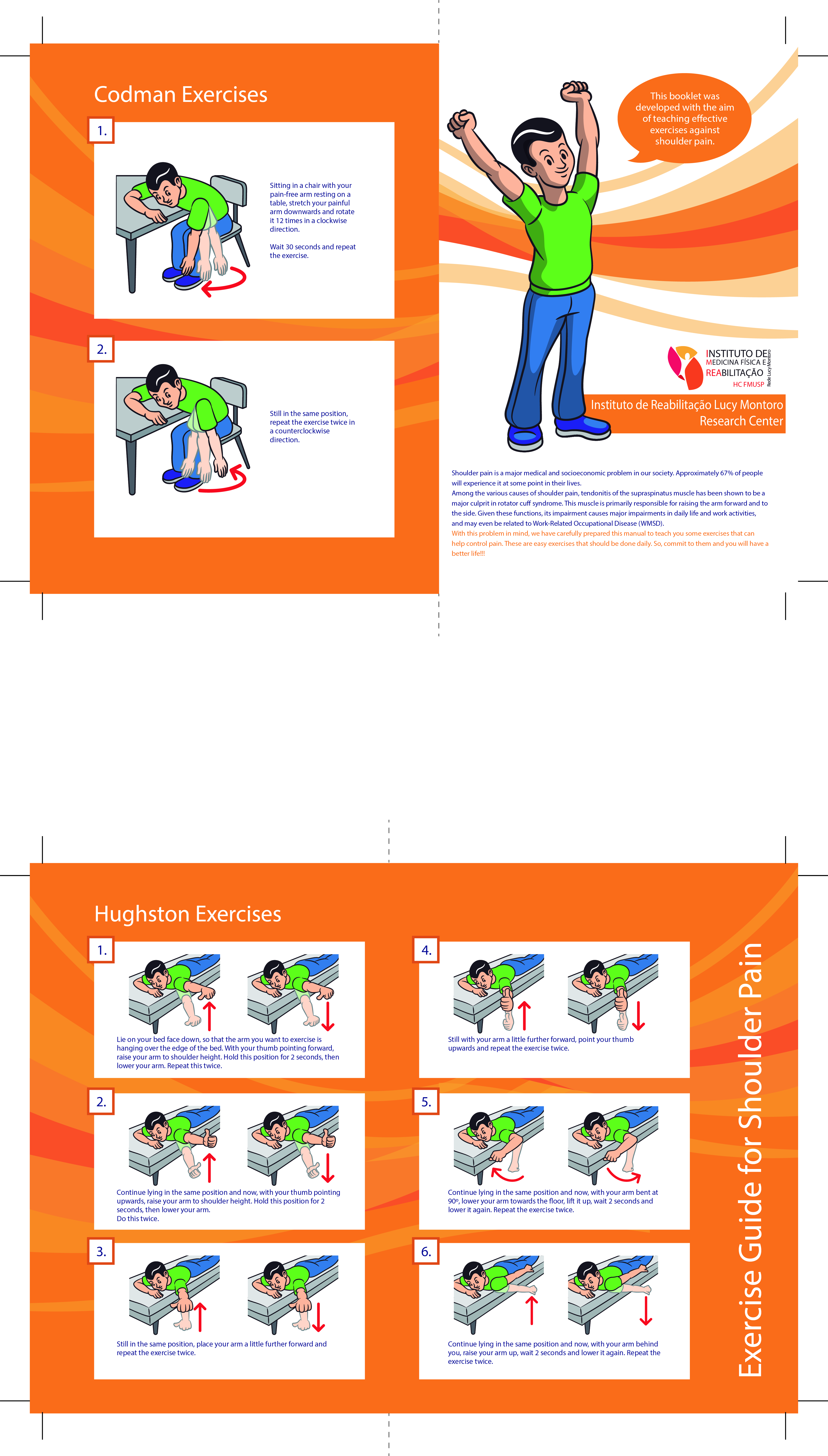

Supplement: Supplementary file 1 [file Image1.jpeg]
